# Baseline of miltefosine resistance markers in zoonotic *Leishmania infantum* from Eastern Mediterranean dogs prior to widespread drug use

**DOI:** 10.1016/j.onehlt.2026.101552

**Published:** 2026-08-18

**Authors:** Georgios Karanikas, Chrysa Voyiatzaki, Daniel Carrasco Navarro, Seray Toz, Maria Antoniou, Stavroula Evi Gouzelou, Despina Smirlis

**Affiliations:** a Molecular Parasitology Laboratory, Hellenic Institute Pasteur, Athens 11521, Greece; b Department of Biomedical Sciences, University of West Attica, 12243 Athens, Greece; c University of Alcala, Spain; d Department of Parasitology, Faculty of Medicine, Ege University, Bornova, Izmir, Türkiye; e Laboratory of Clinical Bacteriology, Parasitology, Zoonoses, and Geographical Medicine, University of Crete, Crete, Greece; f Hellenic Reference Laboratory for Leishmaniasis, Hellenic Pasteur Institute

**Keywords:** *Leishmania infantum*, Miltefosine, Miltefosine sensitivity locus (MSL), Miltefosine transporter (LMT), Canine leishmaniasis

## Abstract

Miltefosine, the only oral antileishmanial drug approved for human use, is increasingly administered in canine leishmaniasis. Reduced susceptibility in *Leishmania infantum* has been linked to alterations in the miltefosine transporter gene (LMT; LINF_130020800) and deletion of the Miltefosine Sensitivity Locus (MSL) on chromosome 31. We analysed LMT presence and MSL integrity by PCR in 66 *L. infantum* isolates collected between 1996 and 2012 from dogs with clinical leishmaniasis in endemic areas of Greece, Cyprus and Türkiye. All isolates carried LMT, indicating presence of the PCR target corresponding to the miltefosine transporter gene. In contrast, 13.6% exhibited MSL deletion (targeting LinJ.31.2370), detected in Greek (33.3%) and Cypriot (9.7%) strains, whereas all Turkish strains retained an intact MSL locus. Given the strong association between MSL deletion and miltefosine treatment failure, these pre-drug-pressure findings provide critical baseline data for the Eastern Mediterranean and underscore the need for molecular surveillance within a One Health framework.

## Highlights

• *Leishmania* miltefosine response is linked to miltefosine transporter gene and sensitivity locus.
• 66 CanL isolates (1996–2012) from Greece (*n* = 18), Türkiye (*n* = 17) and Cyprus (*n* = 31) were analysed.
• All isolates *of Leishmania infantum* isolates retained the miltefosine transporter gene (LMT).
• 13.6% exhibited Miltefosine sensitivity locus (MSL) deletion.
• Regional baseline data support adding MSL status to future resistance surveillance.

## 1 Introduction

Leishmaniases are vector-borne anthropozoonotic diseases caused by protozoa of the genus *Leishmania* that remain a major public-health and veterinary problem in endemic regions. Transmission occurs via phlebotomine sand flies, with parasites alternating between extracellular promastigotes in the insect and intracellular amastigotes in mammalian macrophages. The World Health Organization recognises three main clinical forms—cutaneous, mucocutaneous, and visceral leishmaniasis (VL)—the latter being the most severe and potentially fatal if untreated [1]. In Europe and the Mediterranean, *Leishmania (L.) infantum* is the primary agent of zoonotic VL, with dogs as the main domestic reservoir [2]. The Eastern Mediterranean, including Greece, Cyprus, and Türkiye, is a significant endemic focus with high canine leishmaniasis prevalence [3], [4], [5].

Treatment options “for humans” are limited, including pentavalent antimonials, amphotericin B, paromomycin, and miltefosine [1]. Miltefosine, the first oral antileishmanial, initially showed high efficacy in VL, including antimony-resistant cases, but increasing reports of relapse and treatment failure have raised global concerns about emerging resistance [6], [7]. In Europe, miltefosine (Milteforan®, Virbac) has been licensed for dogs since 2007. In Greece and Cyprus it is frequently used as first-line therapy in dogs in combination with allopurinol, whereas in Türkiye it is often reserved as second-line [8]. Repeated veterinary use exerts drug pressure on the canine reservoir. Because canine visceral leishmaniasis is a zoonosis, selection of resistant parasites in dogs may favour the emergence and dissemination of resistant strains and thereby increase the risk of transmission of resistant parasites to humans [9].

Miltefosine acts via multiple mechanisms, including disruption of membrane lipid metabolism, mitochondrial dysfunction, and interference with phospholipid translocation. A key resistance marker is the Miltefosine Sensitivity Locus (MSL) on chromosome 31, whose deletion strongly associates with treatment failure [10]. The MSL contains genes (including LinJ.31.2370 encoding 3′-nucleotidase/nuclease) that are critical for susceptibility; its deletion occurs via recombination between flanking direct repeats and has been functionally validated by experimental complementation and loss-of-function assays demonstrating a direct contribution to the miltefosine-resistance phenotype [6], [11], [12].

The miltefosine transporter (LMT; LINF_130020800), a P-type ATPase responsible for inward translocation of the drug, is another major molecular marker of miltefosine susceptibility. Inactivating mutations or loss of LMT (often together with its beta-subunit Ros3) markedly reduce drug uptake and confer resistance in both laboratory and clinical isolates [13].

While MSL deletions have been extensively documented in Brazil [6], [11], [12], their prevalence in Old World L. *infantum* populations, particularly in the Eastern Mediterranean, remains largely unknown prior to intensified veterinary miltefosine use. Many of the isolates analysed here pre-date widespread miltefosine deployment (1996–2012), offering a unique pre-drug-pressure baseline essential for future surveillance. Because MSL deletions and LMT alterations can arise independently via recombination “or mutation” and may be selected under drug pressure, their emergence carries direct implications for both animal welfare and public health in zoonotic settings [11], [14].

In this study, we screened *L. infantum* isolates from dogs in Greece, Cyprus, and Türkiye for LMT and MSL integrity to establish baseline prevalence of these resistance-associated markers and to support evidence-based One Health monitoring strategies.

## 2 Materials and methods

### 2.1 Parasite isolates and DNA extraction

A total of 66 *L. infantum* isolates were included, all obtained from dogs with clinical leishmaniasis in endemic areas of Greece, Cyprus and Türkiye. Isolates were selected from established cryobanks on the basis of confirmed species identity (*L. infantum*), availability of high-quality DNA, and geographic representation of the three countries; many had been previously characterised [15], [16], [17]. Ethical approvals were obtained for the use of the *Leishmania* biobank (Pasteur Ethical Committee no. 3079/10.6.2026).

All isolates were maintained under controlled culture conditions and underwent fewer than 10 in vitro passages before DNA extraction, thereby minimising culture-induced genetic or epigenetic drift.

### 2.2 PCR detection of the LMT and the MSL locus

The presence of the miltefosine transporter gene (*LMT*; LINF_130020800) and the miltefosine sensitivity locus (MSL) was assessed by conventional PCR. MSL integrity was evaluated indirectly by amplification of the internal gene *LinJ.31.2370*, whose absence correlates with deletion of the entire locus(Carnielli et al., 2019).

A 222-bp fragment of *LMT* was amplified using primers LMT-F (5′-CAAGTGCCTTTCCACCAGAATC-3′) and LMT-R (5′-CTCACCTTTTTGAACTCCAACAGG-3′). MSL was assessed using primers MSL-F (5′-ATCTAGATTATAAATCCAGTGCGATCG-3′) and MSL-R (5′-TATAAGCTTCTGTCATCACTCTTGTTAATGCG-3′) targeting *LinJ.31.2370*. The α-tubulin gene was amplified as an internal control to verify DNA quality using primers α-tub-F (5′-AGCTGTCCGTCGCGGACATCACGAACTCGGTGTTT-3′) and α-tub-R (5′-CGAACTGAATTGTGCGCTTCGTCTTGATCGTCGCAAT-3′).

## 3 Results

### 3.1 PCR detection of the LMT and the MSL locus

**Fig. 1:**
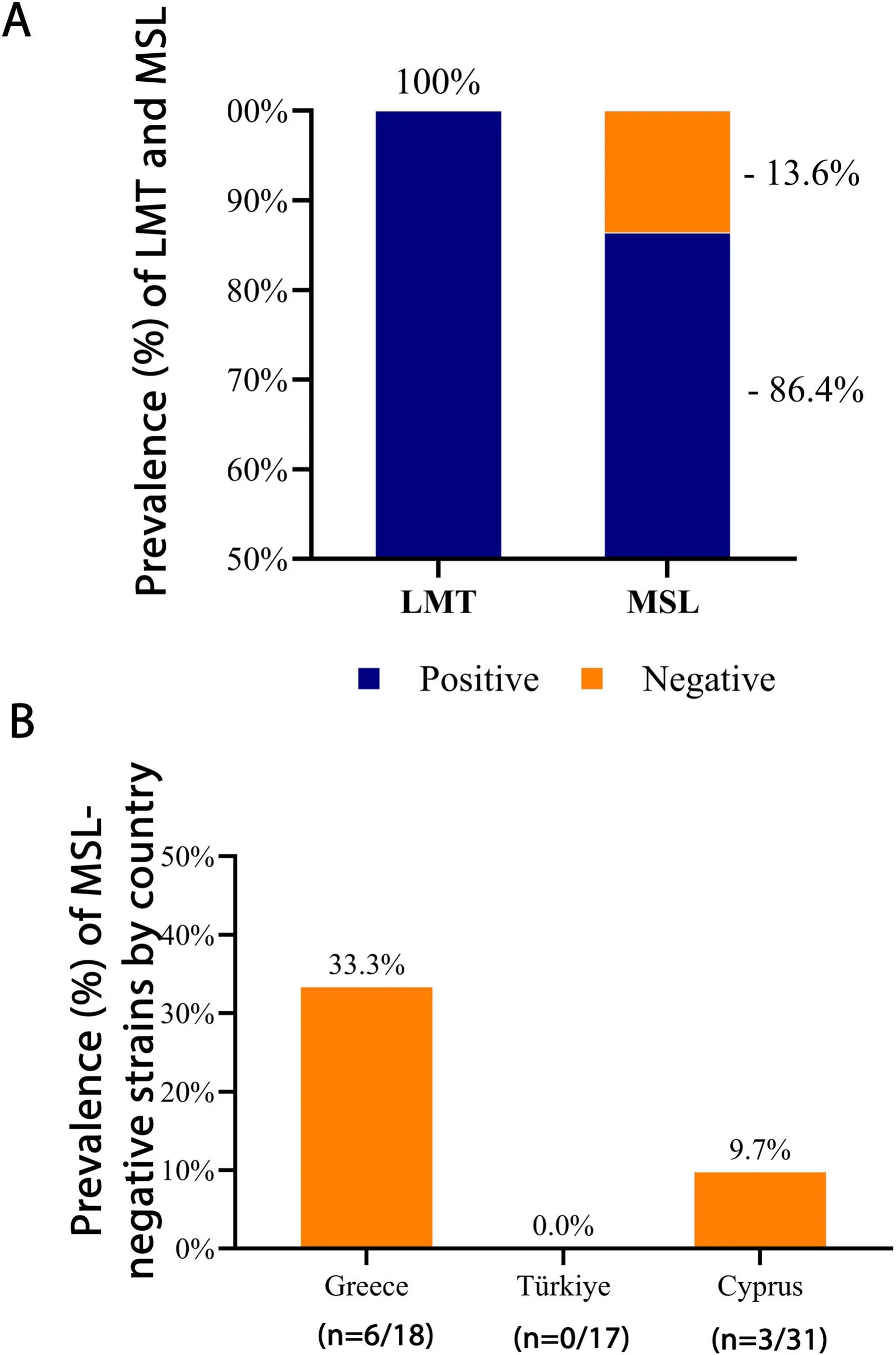
Prevalence of LMT and MSL in canine *L. infantum* isolates from the Eastern Mediterranean and frequency of MSL-negative isolates by country. (A) Bars show the proportion of 66 canine *L. infantum* isolates that were positive or negative for the miltefosine transporter gene LMT (LINF_130020800) and for the miltefosine sensitivity locus (MSL, assessed by PCR amplification of LinJ.31.2370). Values are expressed as percentage of isolates per marker (LMT present/absent, MSL present/absent). All isolates were LMT-positive, whereas a subset lacked the MSL amplicon and were classified as MSL negative. Y-axis: Prevalence (%) of LMT and MSL. (B) Percentage of canine *L. infantum* isolates with MSL-negative PCR result among samples from Greece, Cyprus and Türkiye. Bars represent the proportion of MSL-negative isolates within each country; labels above the bars indicate the corresponding percentage and the total number of isolates analysed per country (n). Below the x-axis the absolute numbers are shown as Greece (6/18), Cyprus (3/31), Türkiye (0/17). MSL status was determined by PCR amplification of LinJ.31.2370, with isolates lacking the expected 1423 bp product but positive for α-tubulin classified as MSL negative. Y-axis: Prevalence (%) of MSL negative strains by country.

All 66 isolates (18 Greek, 17 Turkish, 31 Cypriot) amplified the expected 222-bp fragment of LMT (LINF_130020800). α-tubulin internal control confirmed DNA quality in all samples (Fig. 1, Table 1).

**Table 1:** Characteristics of East Mediterranean L. *infantum* strains derived from dogs and descriptive analysis of the presence of the LMT and integrity of the MSL locus.

Isolate ID	Country	Region	Year	WHO code	LMT PCR	MSL PCR
GD3	Greece	Crete - Heraklion	2001	MCAN/GR/2001/GD3	Positive	Positive
GD5	Greece	Crete - Lasithi/Sitia	2001	MCAN/GR/2001/GD5	Positive	Positive
GD8	Greece	Crete - Rethymno	2001	MCAN/GR/2001/GD8	Positive	Positive
GD14	Greece	Crete - Heraklion	2003	MCAN/GR/2003/GD14	Positive	Positive
GD26	Greece	Crete - Lasithi/Sitia	2004	MCAN/GR/2004/GD26	Positive	Negative
GD27	Greece	Crete - Lasithi/Sitia	2004	MCAN/GR/2004/GD27	Positive	Negative
GD28	Greece	Crete - Heraklion	2005	MCAN/GR/2005/GD28	Positive	Positive
GD36	Greece	Crete- Lasithi/Sitia	2005	MCAN/GR/2005/GD36	Positive	Negative
GD38	Greece	Crete - Lasithi/Sitia	2005	MCAN/GR/2005/GD38	Positive	Positive
GD64	Greece	Crete - Lasithi/Sitia	2005	MCAN/GR/2005/GD64	Positive	Positive
GD67	Greece	Crete - Lasithi/Sitia	2005	MCAN/GR/2005/GD67	Positive	Negative
GD69	Greece	Crete - Lasithi/Sitia	2006	MCAN/GR/2006/GD69	Positive	Negative
GD99	Greece	–	2006	MCAN/GR/2006/GD99	Positive	Positive
GD100	Greece	–	2006	MCAN/GR/2006/GD100	Positive	Positive
GD104	Greece	Crete-Heraklion	2006	MCAN/GR/2006/GD104	Positive	Positive
GD127	Greece	–	2007	MCAN/GR/2007/GD127	Positive	Positive
GD167	Greece	Thessaly-Trikala	2008	MCAN/GR/2008/GD167	Positive	Negative
GD211	Greece	Peloponnese - Argolis	2009	MCAN/GR/2009/GD211	Positive	Positive
EP16	Türkiye	Aegean - Manisa	1996	MCAN/TR/1996/EP16	Positive	Positive
EP43	Türkiye	Aegean - Aydin (Kuşadası)	2000	MCAN/TR/2000/EP43	Positive	Positive
EP44	Türkiye	Aegean - Aydin (Kuşadası)	2000	MCAN/TR/2000/EP44	Positive	Positive
EP55	Türkiye	Aegean - Aydin (Kuşadası)	2000	MCAN/TR/2000/EP55	Positive	Positive
EP57	Türkiye	Aegean - Muğla	2001	MCAN/TR/2001/EP57	Positive	Positive
EP88	Türkiye	Black Sea - Çorum	2003	MCAN/TR/2003/EP88	Positive	Positive
EP126	Türkiye	Aegean - Muğla (Bodrum)	2005	MCAN/TR/2005/EP126	Positive	Positive
EP134	Türkiye	Mediterranean - Hatay (Antakya)	2007	MCAN/TR/2007/EP134	Positive	Positive
EP174	Türkiye	Aegean - Aydin (Kuşadası)	2009	MCAN/TR/2009/EP174	Positive	Positive
EP180	Türkiye	Aegean - Adana	2012	MCAN/TR/2012/EP180	Positive	Positive
EP183	Türkiye	Aegean - Adana	2012	MCAN/TR/2012/EP183	Positive	Positive
EP184	Türkiye	Aegean - Adana	2012	MCAN/TR/2012/EP184	Positive	Positive
EP185	Türkiye	Aegean - Adana	2012	MCAN/TR/2012/EP185	Positive	Positive
EP186	Türkiye	Aegean - Adana	2012	MCAN/TR/2012/EP186	Positive	Positive
EP187	Türkiye	Aegean - Adana	2012	MCAN/TR/2012/EP187	Positive	Positive
EP188	Türkiye	Aegean - Adana	2012	MCAN/TR/2012/EP188	Positive	Positive
EP189	Türkiye	Aegean - Adana	2012	MCAN/TR/2012/EP189	Positive	Positive
CD1	Cyprus	Limassol	2001	MCAN/CY/2001/CD1	Positive	Positive
CD21	Cyprus	Paphos	2004	MCAN/CY/2004/CD21	Positive	Positive
CD34	Cyprus	Limassol	2005	MCAN/CY/2005/CD34	Positive	Positive
CD37	Cyprus	Paphos	2005	MCAN/CY/2005/CD37	Positive	Positive
CD39	Cyprus	Paphos	2005	MCAN/CY/2005/CD39	Positive	Negative
CD40	Cyprus	Paphos	2005	MCAN/CY/2005/CD40	Positive	Positive
CD42	Cyprus	–	2005	MCAN/CY/2005/CD42	Positive	Positive
CD43	Cyprus	Paphos	2005	MCAN/CY/2005/CD43	Positive	Positive
CD44	Cyprus	Paphos	2005	MCAN/CY/2005/CD44	Positive	Positive
CD48	Cyprus	Limassol	2005	MCAN/CY/2005/CD48	Positive	Positive
CD49	Cyprus	Limassol	2005	MCAN/CY/2005/CD49	Positive	Positive
CD50	Cyprus	Paphos	2005	MCAN/CY/2005/CD50	Positive	Positive
CD54	Cyprus	Paphos	2005	MCAN/CY/2005/CD54	Positive	Negative
CD58	Cyprus	Limassol	2005	MCAN/CY/2005/CD58	Positive	Positive
CD59	Cyprus	Limassol	2005	MCAN/CY/2005/CD59	Positive	Positive
CD60	Cyprus	Limassol	2005	MCAN/CY/2005/CD60	Positive	Positive
CD61	Cyprus	Limassol	2005	MCAN/CY/2005/CD61	Positive	Positive
CD62	Cyprus	Paphos	2005	MCAN/CY/2005/CD62	Positive	Negative
CD66	Cyprus	Nicosia	2005	MCAN/CY/2005/CD66	Positive	Positive
CD68	Cyprus	Paphos	2005	MCAN/CY/2005/CD68	Positive	Positive
CD74	Cyprus	Famagusta	2006	MCAN/CY/2006/CD74	Positive	Positive
CD76	Cyprus	–	2006	MCAN/CY/2006/CD76	Positive	Positive
CD77	Cyprus	Paphos	2006	MCAN/CY/2006/CD77	Positive	Positive
CD78	Cyprus	Paphos	2006	MCAN/CY/2006/CD78	Positive	Positive
CD83	Cyprus	Nicossia	2006	MCAN/CY/2006/CD83	Positive	Positive
CD87	Cyprus	Paphos	2006	MCAN/CY/2006/CD87	Positive	Positive
CD88	Cyprus	Paphos	2006	MCAN/CY/2006/CD88	Positive	Positive
CD90	Cyprus	Paphos	2006	MCAN/CY/2006/CD90	Positive	Positive
CD91	Cyprus	Paphos	2006	MCAN/CY/2006/CD91	Positive	Positive
CD95	Cyprus	Limassol	2006	MCAN/CY/2006/CD95	Positive	Positive
CD108	Cyprus	Larnaca	2006	MCAN/CY/2006/CD108	Positive	Positive

### 3.2 Assessment of the presence of the MSL locus

MSL deletion (absence of LinJ.31.2370 amplicon) was observed in 9/66 isolates (13.6% overall). This variant was present in 6/18 Greek strains (33.3%) and 3/31 Cypriot strains (9.7%), while all 17 Turkish isolates retained an intact MSL (Fig. 1, Table 1). The nine MSL-negative isolates were collected between 2004 and 2008 (Greece: 2004–2008; Cyprus: 2005).

## 4 Discussion

In this study, all canine *L. infantum* isolates from the Eastern Mediterranean yielded a positive PCR amplicon for the miltefosine transporter gene (LMT), while 13.6% displayed deletion of the Miltefosine Sensitivity Locus (MSL). The universal presence of LMT suggests that complete transporter loss is rare in this pre-drug-pressure population (1996–2012), consistent with reported fitness costs associated with LMT/Ros3 disruption [18]. Nevertheless, PCR detection of the gene does not guarantee its expression or functional integrity; point mutations, copy-number variation or epigenetic silencing can still impair drug uptake. *Leishmania* exhibits high genomic plasticity, and several studies have shown that expression levels of LMT and related transporters can vary substantially under different culture or host conditions [19].

MSL deletion in 13.6% of isolates is noteworthy. Although lower than Brazilian rates (up to ∼50%, [6], [11], [12]) its presence in Greek and Cypriot strains shows it is not confined to the New World. Comparisons with other Old-World regions remain limited; available data from Mediterranean and Middle-Eastern isolates are still scarce (no large-scale MSL-prevalence surveys have been published to date), underscoring the value of the present baseline. The apparent absence of MSL-negative strains among Turkish isolates may reflect differences in parasite population structure, transmission intensity, or historical drug exposure; however, these explanations remain speculative and are not supported by data generated in the present study. Given the modest sample sizes, formal statistical comparison (e.g., Fisher's exact test) did not reach significance after correction for multiple testing, and the observed country differences should therefore be interpreted with caution.

Given chromosome 31's repeat-rich, tetrasomic nature, recurrent MSL deletions via recombination are plausible and can emerge under selective pressure [11], [14]. Such variants can rapidly dominate under drug pressure, showing inherent resistance potential.

From a One Health perspective, these findings are concerning as dogs are the primary reservoir for zoonotic *L. infantum* transmission to humans. Data correlating MSL deletion with clinical miltefosine treatment failure are now robust: deletion of the locus increases the risk of treatment failure approximately 9.4-fold, is associated with altered lipid metabolism, and may enhance parasite survival inside macrophages [10], [11], [12]. Complementary experimental work has shown that restoration of the 3′-nucleotidase/nuclease gene (LinJ.31.2370) re-sensitises resistant isolates, confirming a direct functional role [11].

Furthermore, MSL deletions could influence parasite fitness beyond drug susceptibility, affecting vector transmission efficiency or adaptation to different canine breeds and immune backgrounds prevalent in the Eastern Mediterranean. Although direct experimental evidence for altered sand-fly competence or host-adaptation linked to MSL status is still limited, changes in surface nucleotide metabolism and membrane lipid composition could theoretically modulate parasite–vector or parasite–macrophage interactions; this remains a hypothesis requiring further investigation.

Pre-existing MSL-deleted subpopulations in the canine reservoir could expand rapidly with increased miltefosine use in veterinary practice, compromising both canine treatment outcomes and human VL control programs. Moreover, clinically “cured” dogs may continue to harbour resistant parasites and remain infectious to sand flies, sustaining the transmission cycle [9].

While MSL deletion is a valuable molecular marker, it is not fully predictive of clinical failure in isolation [10]. Mixed populations (MSL+/MSL–) and additional genetic/epigenetic changes influence overall susceptibility [10]. This study establishes a robust pre-intervention baseline essential for longitudinal surveillance. In particular, the presence of an intact LMT amplicon does not guarantee functional transporter activity. Although all isolates were maintained with fewer than 10 *in vitro* passages, residual culture-associated genetic drift cannot be completely excluded. Systematic MSL screening, susceptibility testing and clinical follow-up offer a practical One Health strategy.

This study has several limitations. PCR-based detection, while practical for large-scale screening, cannot exclude point mutations, copy-number variations, or expression changes that may still impair drug uptake [20]. Future efforts should combine molecular markers with whole-genome sequencing, vector competence studies, and serological surveillance in dogs and humans.

## 5 Conclusion

In conclusion, this pre-drug-pressure survey reveals low but already detectable levels of MSL deletion in Eastern Mediterranean canine *L. infantum*. While the present work is limited to PCR detection of two molecular markers and does not directly assess clinical treatment outcomes, these baseline data provide an essential reference for future surveillance. They highlight the urgency of proactive molecular surveillance and One Health interventions to preserve miltefosine efficacy.

## Declaration of generative AI and AI-assisted technologies in the manuscript preparation process

During the preparation of this work the authors used [@GROK] in order to improve readability and language. After using this tool/service, the authors reviewed and edited the content as needed and take full responsibility for the content of the published article.

## CRediT authorship contribution statement

**Georgios Karanikas:** Writing – review & editing, Writing – original draft, Methodology, Investigation, Formal analysis, Data curation. **Chrysa Voyiatzaki:** Writing – review & editing, Writing – original draft, Supervision, Investigation, Formal analysis, Data curation. **Daniel Carrasco Navarro:** Writing – review & editing, Methodology, Investigation, Data curation. **Seray Toz:** Writing – review & editing, Resources, Investigation. **Maria Antoniou:** Writing – review & editing, Resources, Investigation. **Stavroula Evi Gouzelou:** Writing – review & editing, Methodology, Formal analysis, Data curation. **Despina Smirlis:** Writing – review & editing, Writing – original draft, Supervision, Investigation, Funding acquisition, Formal analysis, Data curation, Conceptualization.

## Consent for publication

Not applicable.

## Ethics approval and consent to participate

Ethical approvals were obtained for the use of the *Leishmania* biobank (Pasteur Ethical Committee no. 3079/10.6.2026). All isolates were obtained from dogs with clinical leishmaniasis under existing ethical frameworks for the respective cryobanks; no additional animal experiments were performed for this study.

## Funding

The study was funded by the Hellenic Pasteur Institute. DCN was a recipient of a PhD Calmette–Yersin studentship from the International Pasteur Network (IPIN). The funders had no role in study design, data collection and analysis, decision to publish, or preparation of the manuscript.

## Declaration of competing interest

The authors declare that they have no competing interests.

## Data Availability

All data generated or analysed during this study are included in this published article (and its supplementary information files, if any). Isolate details are provided in Table 1. Additional information is available from the corresponding author upon reasonable request.
